# Unusual elevation in Entropy but not in PSI during general anesthesia: a case report

**DOI:** 10.1186/s12871-018-0486-8

**Published:** 2018-02-14

**Authors:** Young Sung Kim, Dongik Chung, Seok Kyeong Oh, Young Ju Won, Il Ok Lee

**Affiliations:** Department of Anesthesiology and Pain Medicine, Korea University Guro Hospital, 148 Gurodong-ro, Guro-gu, Seoul 08308 South Korea

**Keywords:** Artifacts, Electroencephalography, General anesthesia

## Abstract

**Background:**

EEG monitoring is useful for determining an adequate level of anesthesia. However it is sometimes interfered by various reasons. We describe a case in which we successfully confirmed the adequate depth of anesthesia by monitoring the patient state index (PSI), which was computed from the SedLine monitor data in Root (Masimo) during general anesthesia. Our case showed unusual elevations in entropy, but not in PSI.

**Case presentation:**

A 34-year-old woman was scheduled for emergency surgery for a left tibial open fracture and a right femoral closed fracture, which were sustained during a traffic accident. Forty-five minutes after intubation, the response entropy abruptly increased up to 100 and state entropy to 91. Despite the absence of other abnormal events, the entropy data led to two types of incorrect decisions. The first was owing to the effect of the EMG and the second was misleading during the surgeon’s hammering. However, PSI from the SedLine monitor seemed to be less influenced by the same events.

**Conclusions:**

In this report, we suggest that the PSI, derived from new-generation SedLine (Root, Masimo) may be a useful parameter for clinically determining the level of sedation. The use of two monitoring devices with different EEG algorithms might be helpful for determining the anesthetic depth and making decisions.

**Electronic supplementary material:**

The online version of this article (10.1186/s12871-018-0486-8) contains supplementary material, which is available to authorized users.

## Background

It is important to maintain adequate anesthetic depth during surgery. Light anesthesia may increase the risk of patient anxiety, stress responses, and awareness during anesthesia [1–3]; deep anesthesia may give rise to not only cardiovascular and respiratory function impairment, but also hemodynamic instability, an increased risk of delayed emergence, post-operative nausea and vomiting, and other post-operative complications [4]. Prior to the development of electroencephalogram (EEG) monitoring, anesthesiologists were required to determine the depth of anesthesia using limited information, including vital signs or the patients’ responsiveness. However, currently, it is possible to quantify anesthetic depth using a monitoring tool that directly assesses the brain’s response to anesthetics. Routine EEG monitoring allows for a more accurate titration of anesthetics during surgery [5]. Commonly used brain EEG monitoring devices include Bispectral Index (BIS) monitor, E-Entropy, Narcotrend, NeuroSENSE, and SedLine [6]. Among these various EEG monitoring devices, in our center, Entropy for EEG monitoring has been widely used because its modules were already integrated to the Datex-Ohmeda monitor and ventilator.

Although Entropy has been shown to have a high predictive power for the loss of consciousness (LOC) or return of consciousness (ROC) in various clinical situations [4], there are some case reports on unexpected predictions using Entropy [7]. In addition to Entropy, other EEG-derived indices may also inaccurately reflect a patient’s consciousness [8, 9]. It is widely known that many factors, such as electromyographic (EMG) activity [10, 11], concomitant use of surgical devices [12], and electrocardiogram (ECG) artifacts [13], can interfere with EEG-based monitoring.

Recently, Masimo launched a new monitoring device called Root. Root is a multi-parametric monitoring and connectivity platform that augments multiple parameters, including brain function monitoring (SedLine®), regional oximetry (O3®), and capnography and gas monitoring. The SedLine function uses the patient state index (PSI) to assess the patients’ levels of sedation and/or depth of anesthesia. PSI was first introduced in the 2000s. The PSA 4000/5000 (Physiometrix, Inc.) and the SedLine (Masimo) monitors are used to estimate the PSI [14]. Caputo et al. [15] pointed out that the SedLine (previous version) did not sufficiently eliminate EMG activity. However, Masimo reported a new generation of SedLine monitors that compute PSI with less influence from EMG activity (SedLine ver 2.1 vs 2.0). The PSIs derived using the newer version had higher areas under curves (AUCs) than that of the previous version with receiver operating characteristic curves that allowed for the discrimination of LOC or ROC. However, no studies have compared the PSI of SedLine/Root and Entropy/Datex-Ohmeda.

We report unusual elevations when using the Datex-Ohmeda Entropy monitor, but not in the PSI derived from the Masimo Root SedLine monitor used during general anesthesia.

## Case presentation

A 34-year-old woman (height = 160 cm; weight = 50 kg) was scheduled for emergency surgery due to an open fracture of her left tibia and closed fracture of her right femur, which were sustained during a traffic accident. She had no past medical history. There were no other organ injuries, including those of the neck, face, thorax, or abdomen. The results from an ECG and chest radiograph revealed no acute or pathologic lesions.

After obtaining informed consent for surgery and anesthesia, the patient entered the operating room and routine monitoring, including capnography, a 3-lead ECG, non-invasive blood pressure monitoring, mechanosensor-neuromuscular transmission (M-NMT) module, and Entropy module on Datex-Ohmeda, was performed. Her initial blood pressure (BP) was 120/70 mmHg, heart rate (HR) was 93 beats/min, and her pulse oximetry oxygen saturation (SpO_2_) was 99%. After preoxygenation with 100% O_2_ at 8 L/min delivered through a mask for a few minutes, a remifentanil infusion (0.02 mcg/kg/min) was started and 80 mg of propofol was administered intravenously. After LOC, supramaximal stimulation using NMT was checked; thereafter, 35 mg of rocuronium was injected. Tracheal intubation using a cuffed 7.0-mm endotracheal tube was performed without difficulty. Anesthesia was maintained with 1.5 L/min of O_2_, 1.5 L/min of medical air, 1.0 to 2.0 vol% of sevoflurane, and a 0.02 - 0.10 mcg/kg/min remifentanil infusion. Thirty minutes after intubation, surgical drapes and other preparations were completed and an incision was performed. Her vital signs and other monitored parameters were within a 20% range of the preoperative levels.

**Fig. 1:**
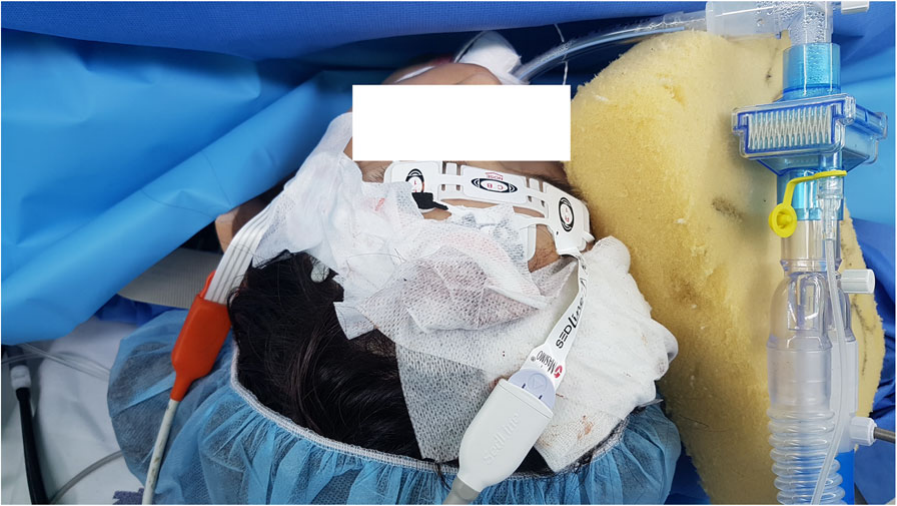
The application of Entropy and SedLine sensors. To perform additional SedLine EEG monitoring, we moved the Entropy disposable sensor to the upper forehead

**Fig. 2:**
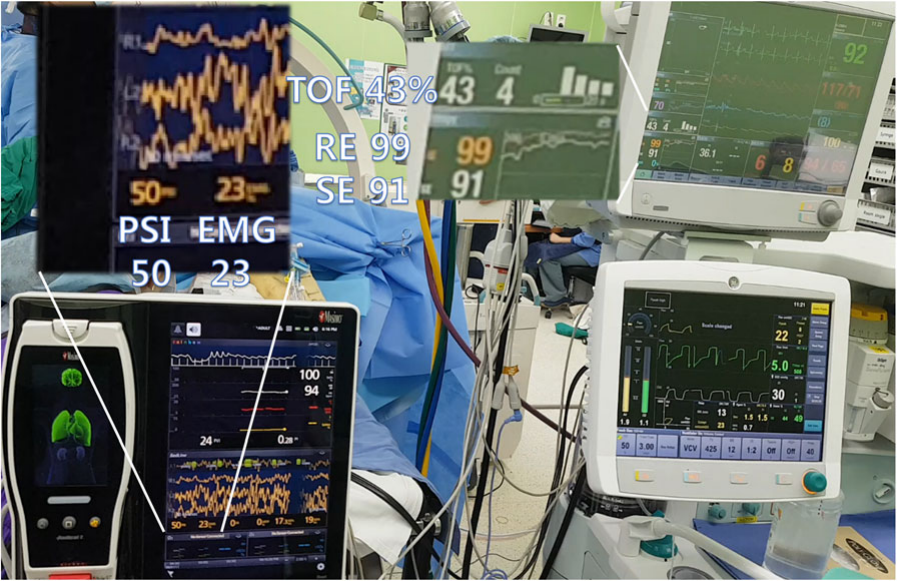
The first case of unusual elevation on Entropy by EMG activity, but not on PSI. Ninety-nine of response entropy (RE) and 91 of state entropy (SE) were assessed, while 50 of patient state index (PSI) was assessed. 43% of train of four (TOF) on NMT and 23% of EMG on SedLine were assessed at the same time

**Fig. 3:**
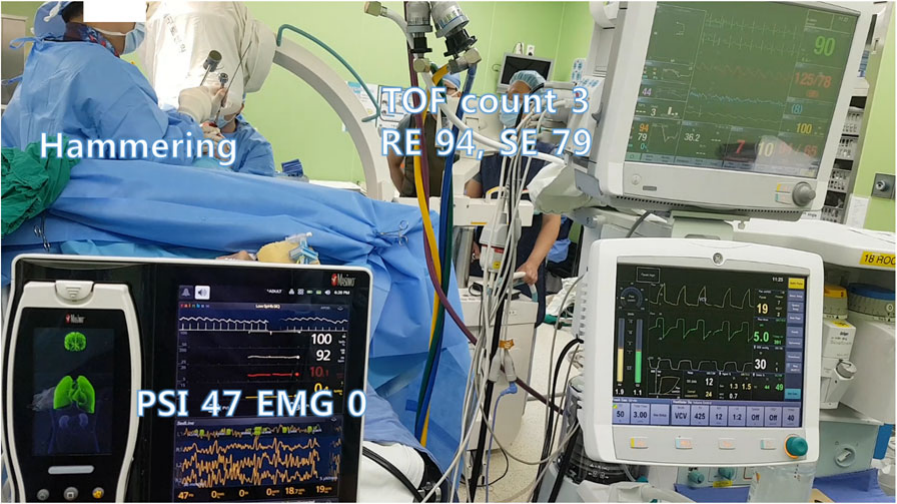
The second case of unusual elevation on Entropy by use of surgical device (constant hammering), but not on PSI. Ninety-four of response entropy (RE) and 79 of state entropy (SE) were assessed, while 47 of patient state index (PSI) was assessed. Three counts of train of four (TOF) on NMT and 0% of EMG on SedLine were assessed simultaneously

However, 45 min after intubation, the response entropy (RE) abruptly increased up to 100 and the state entropy (SE) to 91. NMT was exhibited in 4 of the counts with a 46% train of four (TOF) ratio, even though capnography showed no sign of spontaneous breathing. There were no significant changes in her vital signs. Even though 3 vol% of sevoflurane was temporarily administered, the entropy did not change. Because there was no other evidence of ROC, we applied additional EEG monitoring and used the Root with SedLine monitor (Fig. 1) and confirmed a PSI of 50% and EMG of 23% (Fig. 2). We assumed that the elevated EMG activity might cause an unusual elevation in the readings from the Entropy monitor. Thereafter, 10 mg of rocuronium was injected. After a few tens of seconds, TOF of NMT and RE/SE values on the Entropy monitor decreased gradually and simultaneously. When the NMT count reached 2 (2 mins after rocuronium injection), 59% of RE, 58% of SE, 40% of PSI, and 0% of EMG were assessed. Coincidently, the surgeon started to hammer on the nail in the patient’s tibia. He hammered regularly using the continuously repeated “hit - hit - pause” method. The “pause” step occurred at approximately 1.8 Hz of frequency. Within a minute, 3 TOF counts of NMT, 94% of RE, 79% of SE, 47% of PSI, and 0% of EMG were assessed (Fig. 3). However, after a few minutes, the value of entropy decreased by less than 60. Twenty minutes later, the TOF count reached 4 and RE/SE values on the Entropy monitor increased to above 90 again while the PSI values were maintained below 50. Therefore, we discontinued monitoring with Entropy.

No other specific events occurred during the remaining intraoperative period. The patient did not complain of recall in the postoperative period. Upon discharge from the PACU, the last memories the patient had prior to sleep were the events of the surgical time-out just after entering the operating room. The first memory the patient had after awakening was the instruction from the medical staff to open her eyes in the recovery room. The patient did not dream during surgery. Mild surgical pain and dry mouth were the only discomforts the patient complained of in the recovery room.

## Discussion and conclusions

EEG monitoring techniques have markedly evolved since the introduction of BIS monitoring in 1996 [16]. EEG monitoring helps maintain an adequate depth of anesthesia, which leads to a sparing effect of the anesthetics [16, 17]. While other EEG monitoring systems have not revealed their algorithm, the Entropy algorithm has been made available to the public [18]. When considered as a physical concept, entropy indicates “disorder” in the system. In the case of EEG signals, entropy describes the irregularity, complexity, or unpredictability of the signal. Meanwhile, Fourier’s theorem revealed that all periodic functions can be represented as the sum of simple sine waves. That is, any EEG signal can be disassembled to many sine waves by their own frequencies. Each frequency has power, which individually contributes to the entire wave. If only one frequency has 100% power, indicating a perfect sine wave, there is no irregularity. Therefore, entropy is zero. On the contrary, if every frequency contributes an even amount of power, this creates the maximum irregularity and entropy is 100%.

The next step is to distinguish the EEGs from the all the signals. Fortunately, we already know the EEG dominant frequency. The EEG-dominant portion of the spectrum ranges from 0.8 Hz to 32 Hz. There is overlap from 32 Hz to 47 Hz, whether it be EEG-dominant or EMG-dominant. A frequency greater than 47 Hz is useless when interpreting the EEG. When “0.8 Hz to 47 Hz” is set as the maximum (100), “0.8 Hz to 32 Hz” naturally becomes 91 [18].

In our case report, there were two types of artifacts contributing to the unusual elevation in entropy.

The first was an incorrect analysis of the EMG. Unlike the theory, entropy failed to discern the EMG component. In 2014, we already reported a false elevation of the bispectral index due to EMG activity and signal interference from the pulsation of the carotid artery [19]. In that case, the frequency of the carotid artery pulsation was 1.5 Hz (90 of pulse rate), which is within the EEG range (0.8 to 32 Hz) [18]. In increased EMG circumstances, the power of the wave corresponding to the frequency matching the pulse may be over-amplified. However, this case is somewhat different from the previous one. In our case, EMG itself may have been misinterpreted as EEG [10, 11].

The second was concomitant use of surgical devices. The Entropy module might misinterpret the constant hammer of 1.8 Hz as EEG signals. In the second situation, the effects of EMG were excluded by use of rocuronium just before observation and a confirmation of 0% EMG activity on the SedLine module. Both artifacts coincidentally developed during a short period of time. Details can be found in the attached video (Additional file 1).


**Additional file 1: Movie S1.** 00:00.00 RE 96, SE 91, TOF 44% (Right upper), PSI 48, EMG 20 (Left). 00:04.93 Rocuronium 10 mg injection (Left). 00:57.98 Entropy values are decreasing (Right upper). 01:19.10 EMG 0% checked (Left). 01:41.93 RE 48, SE 46, TOF 5% (Right upper), PSI 42, EMG 0 (Left). 02:30.00 Start hammering (Left upper). 03:21.81 Entropy started to increase, TOF abruptly increased: 0 to 56% (Right upper), no change on PSI, EMG (Left). 03:26.71 Entropy increasing with constant hammering. 03:50.10 RE 97, SE 79, TOF count 3 (Right upper), PSI 47, EMG 0 (Left). 04:00.10 Entropy decreasing by itself (Right upper), no change on PSI, EMG (Left). (MP4 134005kb)


Because the precise algorithms of the SedLine are not disclosed to the public, it is hard to precisely explain how the SedLine excludes these two artifacts. PSI is calculated using proprietary algorithms by a 4-channel EEG monitor after advanced artifact rejection. The parallel signal processing engines of the PSI may have advantages for discriminating EEG waves from other signal noises. It evaluates EEG data according to a coherence between the bilateral regions and anterior-posterior relationships in the brain [20]. The new-generation SedLine also utilizes an adaptive signal processing with band-independent features, which is advantageous in cases of low-power EEG because it searches across many EEG frequency bands. Our findings support the enhanced signal stability of the new-generation SedLine PSI.

There are some limitations in our case report. First, as shown in Fig. 1, the position of the Entropy sensor was higher than that of the SedLine sensor on the forehead. Prior to attaching the SedLine sensor, the Entropy sensor was properly placed. However, in order to attach the SedLine sensor in its place, we had to shift the Entropy sensor up. Although the abnormal elevation of entropy accompanied by an EMG elevation was observed even before the Entropy sensor moved, their different positions are associated with the limitation in an exact comparison between the two EEG monitors. Second, we only provided the trend and values from Entropy and not the raw EEG data from Entropy. To assess the quality of the EEG signal, we should have checked the raw EEG data from the Datex Ohmeda monitor, in addition to the Masimo monitor.

Nevertheless, this has several advantages. To the best of our knowledge, there are no reports of artifacts that occur only one side while assessing two monitors at the same time. Moreover, it is uncommon for two types of artifacts to be observed during a short period of time. The relatively low frequency of constant hammering in our second situation is differentiated to other reports of EEG interferences with surgical devices. Although the probability to be deviated by the EEG score is less likely, the wrong anesthetic depth may give rise to critical complications. Therefore, we think the impact of our findings is not weak.

We do not intend to claim superiority or inferiority of the new SedLine. The evidence of superiority of the Root with SedLine monitor over the Datex Ohmeda Entropy monitor is still lacking. Clinically, the findings here can aid anesthesiologists who are confused about the depth of anesthesia. Our case suggests that implementing two monitoring devices with different EEG algorithms might be helpful for determining anesthetic depth and making decisions. A score generated using an algorithm may occasionally provide the wrong answers; however, with the addition of EMG monitoring and raw EEG data, if the patient and the surgical procedures are well understood and observed, we suggest that EEG monitors can still be useful guides for managing anesthesia.

## Abbreviations

AUCs: Areas under curves
BIS: Bispectral Index
ECG: Electrocardiogram
EEG: Electroencephalogram
EMG: Electromyography
PSI: Patient state index
RE: Response entropy
SE: State entropy
TOF: Train of four
